# Case report: what gives the myopic tilted disc an oval appearance?

**DOI:** 10.1186/s12886-020-1305-9

**Published:** 2020-01-09

**Authors:** Kyoung Min Lee, Martha Kim, Seok Hwan Kim

**Affiliations:** 1 Department of Ophthalmology, Seoul National University College of Medicine, Seoul, South Korea; 2 Department of Ophthalmology, Seoul National University Boramae Medical Center, Seoul, South Korea; 3 Department of Ophthalmology, Dongguk University Ilsan Hospital, Goyang, South Korea

**Keywords:** Myopic tilted disc, Tilt, Ovality, Boramae myopia cohort study

## Abstract

**Background:**

Myopic tilted disc, observed as an oval disc, has been alleged to be a funduscopic en-face manifestation of excessive optic nerve head (ONH) sloping or tilting. Here, we report the case of a myopic child showing a developing oval disc in fundus photos during axial elongation, but without progressive tilting in spectral-domain optical coherence tomography (SD-OCT) images.

**Case presentation:**

By merging B-scan SD-OCT images of the ONH and macula, the curvature of the posterior pole, including both the fovea and ONH, was reconstructed and compared before and after 2 years of axial elongation. Despite the marked increase of disc ovality, the posterior polar curvature was rarely changed. The preponderance of optic disc change was induced by the shift of the temporal disc margin in the nasal direction. This shifting alone imitated an increase of tilt angle but one that was still far smaller than the required degree of tilt for ONH-tilt-based disc ovality. To clarify, we calculated the required extent of axial elongation to obtain a substantial degree of ONH tilt when considering the adjacency of the fovea and the ONH. Without a focal increase of posterior polar curvature, which is to say posterior staphyloma, such change is not possible until the axial length increases extraordinarily.

**Conclusion:**

The most prominent change in the development of myopic tilted disc, which change gives it an oval appearance and imitates a tilt when measured, is actually not a tilt but rather a shift of the temporal disc margin.

## Background

As an eyeball is a sphere, the fundus is a curved plane, and both the optic nerve head (ONH) and fovea are on the surface of that curved plane, with some sloping of the former relative to the latter. Myopic tilted disc has been alleged to be a funduscopic en-face manifestation of excessive ONH sloping or tilting in this curved plane [1–3]. In the Boramae Myopia Cohort Study [4–6], however, we theoretically showed that disc ovality cannot be explained by the aspect of tilt. We found that the inner retinal structure of the posterior polar area including the Bruch’s membrane opening was relatively preserved during axial elongation, while the outer load-bearing structure expanded. This expansion of the sclera and consequent shift of the lamina cribrosa from the preserved Bruch's membrane opening may result in the change of ONH shape that is seen in myopia [4–6]. Herein, we report a case developing oval disc in fundus photos, but without progressive tilting in spectral-domain optical coherence tomography (SD-OCT) images.

## Case presentation

**Fig. 1:**
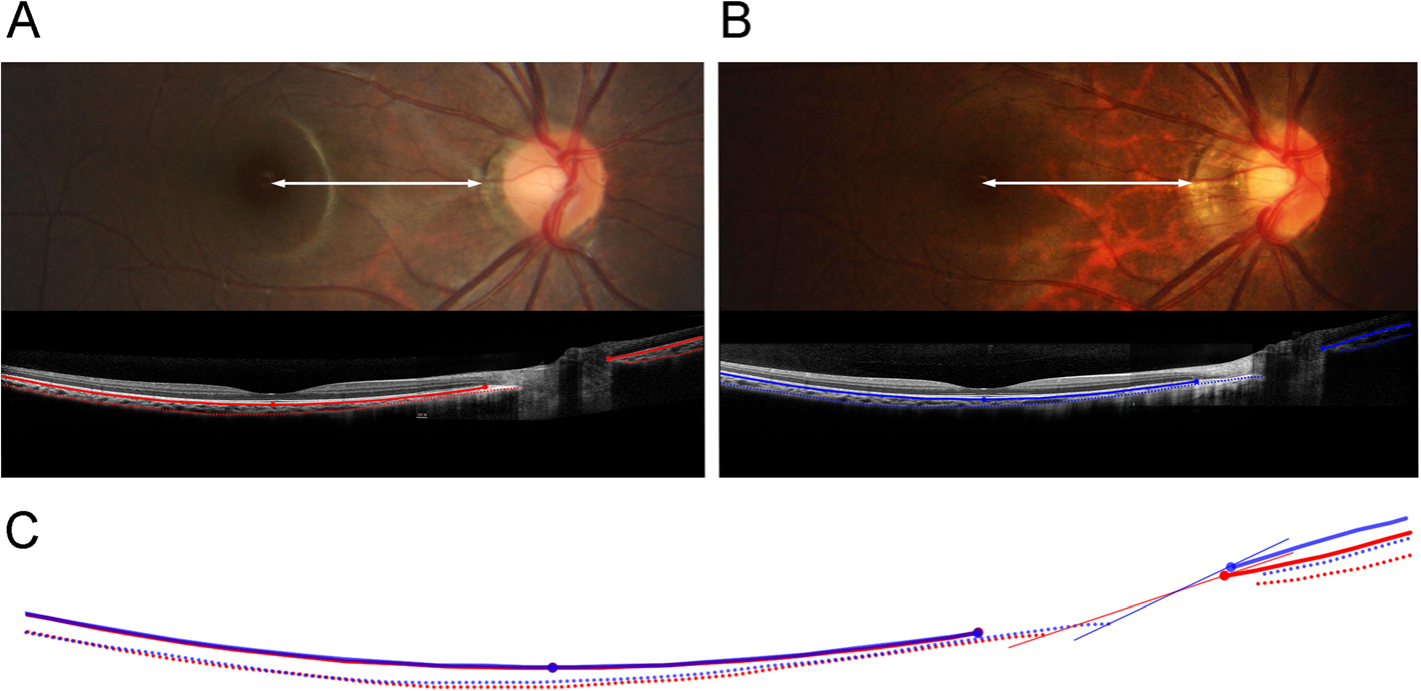
The optic disc has changed from the initial (**a**) to the final (**b**) visit. The distance from the fovea to the temporal margin of parapapillary atrophy was unchanged (white double arrows of same length), while the γ-zone was elongated. The horizontal diameter of this optic disc had been reduced to 0.772 of its original size. To account for the optic disc change of this patient, 39.5° of tilt by arccosine (0.772) was required. The curvatures along the Bruch’s membrane (linear lines) and anterior sclera (dotted lines) are drawn at both the initial (red lines) and final (blue lines) visits. The merged image (**c**) shows that the degree of tilting or sloping of the optic nerve head (ONH) is negligible, while the majority of optic disc change is induced by the elongation of the scleral layer uncovered by the Bruch’s membrane: γ-zone parapapillary atrophy. The ONH tilt angle of initial and final visits were 5.4° and 9.8°, respectively, when measured by the method described in: Marsh-Tootle WL, Harb E, Hou W, Zhang Q, Anderson HA, Weise K, Norton TT, Gwiazda J, Hyman L: Optic Nerve Tilt, Crescent, Ovality, and Torsion in a Multi-Ethnic Cohort of Young Adults With and Without Myopia. *Invest Ophthalmol Vis Sci* 2017, 58(7):3158–3171

This girl had been enrolled in the Boramae Myopia Cohort Study when she was 8 years old, and was observed for 2 years. Her best-corrected visual acuity was 20/20 in the right eye throughout the entire study period, while her refractive error worsened from − 7.5 Dsph = − 0.50 Dcyl × 180 A to − 8.75 Dsph = − 1.50 Dcyl × 180 A. During the same period, her axial length increased from 24.62 mm to 25.7 mm, and the horizontal optic disc diameter had been reduced to 0.772 of its original size (Fig. 1). If tilting or sloping was the reason, its’ angle should have been 39.5° by arccosine (0.772) [5]. Here, to show the change of posterior polar curvature, we merged the B-scan SD-OCT images of the macula and optic nerve head. Except for small differences in the nasal curvature, the posterior polar curvatures of Bruch’s membrane and the anterior sclera were nearly identical between the initial and final visits (Fig. 1c). There was almost no progressive ONH tilting or sloping of the ONH. The majority of optic disc change was induced by the nasal elongation of the scleral layer uncovered by the Bruch’s membrane: γ-zone parapapillary atrophy.

## Discussion

**Fig. 2:**
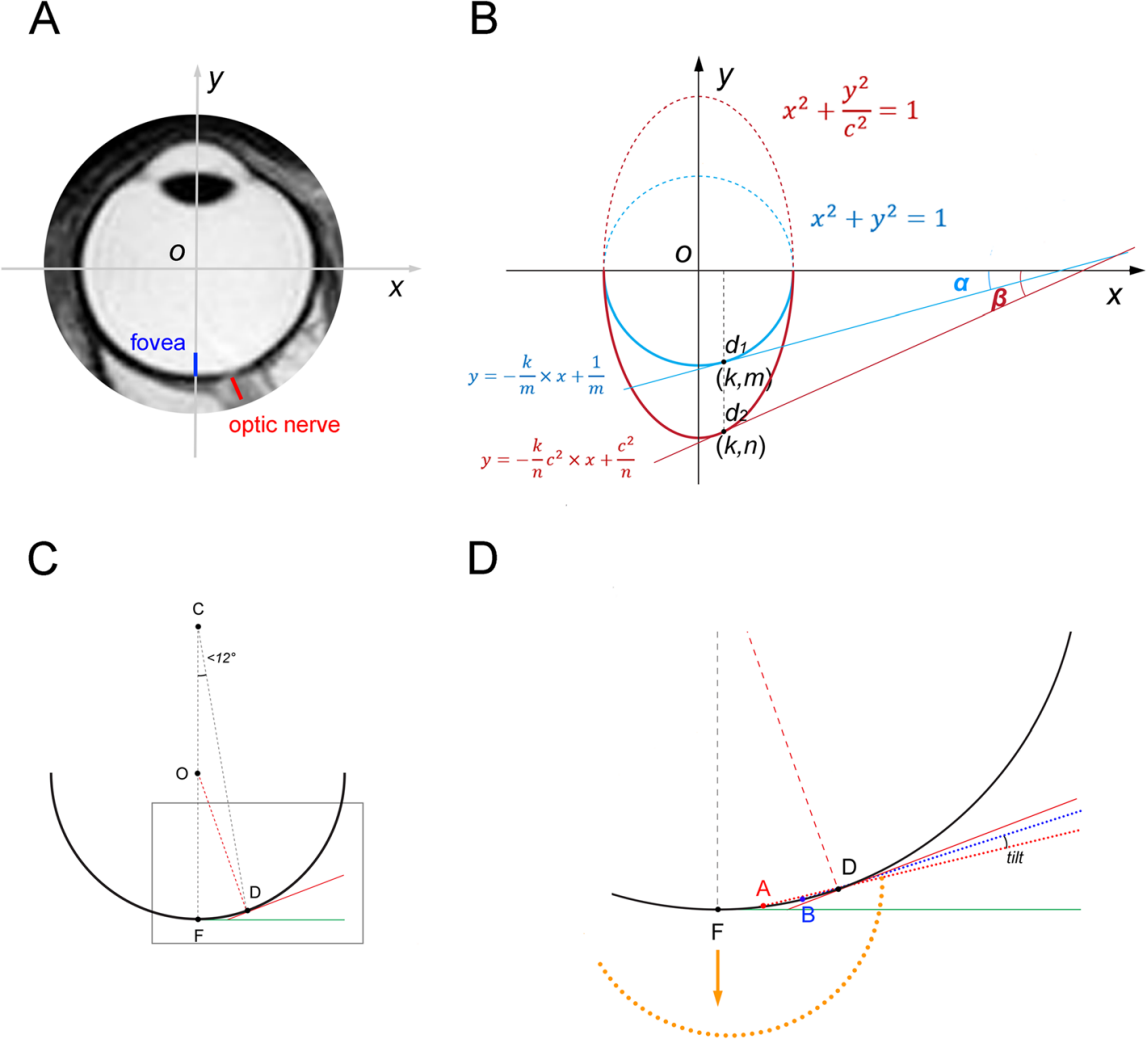
The distance between the fovea and the optic disc, which generates sloping of the optic disc. **a** T2-weighted MRI image of eyeball. Please note the actual distance from the fovea (blue line) to the optic disc (red line). The *x* and *y* axes are drawn to simulate axial growth in the rectangular coordinate system. **b** The axial growth is simulated by the c times increase of a unit sphere along the *y* axis. In order to simplify the calculation, the optic disc locations before and after the axial elongation are designated as *d*_*1*_ and *d*_*2*_, respectively. The optic nerve head (ONH) tilt angle can be defined as the angular deviation of the tangential lines at the disc points (*d*_*1*_ and *d*_*2*_) from the *x* axis (*α* and *β*). From the equations of a sphere and an ellipsoid, the equations of tangential lines at *d*_*1*_ and *d*_*2*_ could be calculated (Appendix). Since the ONH is very close to the fovea relative to the horizontal diameter of the eye, $\frac{\beta }{\alpha}\approx \frac{\tan \beta }{\tan \alpha }=c$ (Appendix). This means that, for the initial ONH tilt angle of *α* to be 2 *α*, the additional ONH tilt of angle *α* requires a 2-fold increase of the eyeball’s diameter along the *y* axis (*c* = 2). This degree of tilt would not be acquired until the axial length has increased from 24 to 36 mm, even if we assume that axial growth occurs only in the posterior half of the eyeball. We could hardly observe either significant sloping of the emmetropic optic disc or high myopia with an axial length of 36 mm without any posterior staphyloma. **c**, **d** Shifting of the temporal disc margin, which imitates progressive tilt despite the same posterior polar curvature. **c** Let us imagine a best fit sphere along the posterior polar curvature. F is the fovea, and D is the nasal optic disc border. These two points are within 12° in the external view, when we considering that the Humphrey visual field test 24–2 can capture the blind spot in nearly all cases. The tangential lines at point F (green line) and at point D (red line) are drawn. **d** Magnified view between the fovea and the ONH. If the temporal optic disc border is shifted from A to B, the angle becomes more oblique (red dotted line and blue dotted line). Please note that the shifting is exaggerated to visualize the angular difference. Not only is the angular difference small, but also, the angle cannot exceed the angle of the tangential line at point D (red line), regardless of how large the shift that occurs is. The only exception is the posterior staphyloma (orange dotted line), which has a focally steeper change of curvature in the posterior pole. In this case, the ONH can be truly tilted

The absence of progressive tilting is obvious when we consider how closely the ONH and the fovea are located in comparison with the whole eyeball size on MRI (Fig. 2a). The ONH is very close to the fovea (within 12° from the external view) [7] relative to the horizontal diameter of the eye. If axial growth is modeled mathematically in the rectangular coordinate system, the ONH tilt angle could be back-calculated from tangential lines at the points of the optic disc (*d*_*1*_ and *d*_*2*_) of a sphere and an ellipsoid (Fig. 2b), which reveals that a unit sphere has to be elongated 2 times for the initial tilt angle of *α* to be 2 *α* (Appendix). This means that this degree of tilt would not be acquired until the axial length has increased from 24 to 36 mm, even if we assume that axial growth occurs only in the posterior half of the eyeball. The disc tilt angle (angle *α*) of emmetropic eyes was reported to be 2.4° in a multicenter cohort study [3]. Therefore, even extraordinary growth can produce only a negligible degree of additional tilt in this axial growth model.

Moreover, many myopic eyes have a growth pattern that is focused on the equatorial region while preserving the posterior curvature [8, 9]. This would make optic disc tilt almost impossible. Instead, we think that shifting of the temporal disc margin might imitate progressive tilting (Fig. 1c). During axial elongation, the temporal ONH border is rotated from internally oblique to externally oblique, and then, the externally oblique border is elongated nasally [4–6]. In the area of the externally oblique border, the temporal disc margin is reported to coincide with the anterior scleral opening [10]. Therefore, increased disc ovality is related to nasal shifting of the temporal disc margin. Theoretically, a tilt angle [3] increases as a result of a temporal disc margin shift (from point A to point B in Fig. 2d, shown exaggeratedly) and a stable nasal disc margin (point D in Fig. 2d). This is due to the fact that the angle would be measured more obliquely on the periphery of the curvature (Fig. 2d). In our case, the disc margin shift alone imitated a tilt of 4.4° (Fig. 1c), similar to the tilt angle difference of 3.6° between myopia and emmetropia groups in a multicenter study [3]. More tilt will necessitate a steeper curvature (=smaller radius) in the posterior pole: posterior staphyloma (Fig. 2d, orange dotted line) [8].

## Conclusions

In describing myopic oval disc thus far, we have been preoccupied by the concept of tilt and have pointed to the eyeball’s spherical shape as a cause. However, almost no progressive change of posterior polar curvature was observed despite huge change of disc ovality, and considering the adjacency of the fovea to the optic disc, the ONH tilt angle cannot exceed a very limited range. Therefore, the most prominent change in development of myopic oval disc is not a tilt rather a shift.

## Data Availability

The data supporting the conclusions of this article are contained within the manuscript. The datasets used and/or analyzed during the current study are available from the corresponding author on reasonable request.

## Appendix

Let us imagine a unit sphere and an ellipse elongated *c* times along the *y* axis to simulate prolate axial growth.

The equations of the unit sphere (blue line) and the ellipse (red line) are

$$ {x}^2+{y}^2=1\  and\ {x}^2+\frac{y^2}{c^2}=1 $$

The optic disc locations before and after the axial elongation are designated as *d*_*1*_ and *d*_*2*_, respectively. Since the foveo-Bruch’s membrane distance is preserved during axial elongation, *d*_*1*_ and *d*_*2*_ would share the same *x* values: (*k*, *m*) and (*k*, *n*).

The tangential line at *d*_*1*_ is:

$$ x\times k+y\times m=1 $$

$$ \therefore y=-\frac{k}{m}\times x+\frac{1}{m} $$

The tangential line at *d*_*2*_ is:

$$ x\times k+\frac{y\times n}{c^2}=1 $$

$$ \therefore y=-\frac{k}{n}{c}^2\times x+\frac{c^2}{n} $$

Therefore,

$$ -\frac{k}{m}=\tan \alpha, -\frac{k}{n}{c}^2=\tan \beta $$

$$ \therefore \frac{\tan \beta }{\tan \alpha }=\frac{m}{n}\times {c}^2 $$

Now, since *d*_*1*_ (*k*, *m*) and *d*_*2*_ (*k*, *n*) are on the sphere and ellipse, respectively,

$$ {k}^2+{m}^2=1,{m}^2=1-{k}^2 $$

$$ {k}^2+\frac{n^2}{c^2}=1,{n}^2={c}^2\times \left(1-{k}^2\right) $$

$$ \therefore \frac{m^2}{n^2}=\frac{\left(1-{k}^2\right)}{c^2\times \left(1-{k}^2\right)}=\frac{1}{c^2},\frac{\tan \beta }{\tan \alpha }=\frac{m}{n}\times {c}^2=c $$

If |*x*| ≪ 1, we can approximate tan *x* to *x*, because the Taylor series expansion for the tangent function is as follows.

$$ \tan x=x+\frac{1}{3}{x}^3+\frac{2}{15}{x}^5+\frac{17}{315}{x}^7+\cdots $$

The ONH is very close to the fovea relative to the horizontal diameter of the eye (*k* ≪ 1 ).

∴|*k*| ≪ |*m*| < |*n*|, and |tan*α*| < |tan*β*| ≪ 1

$$ \therefore \frac{\beta }{\alpha}\approx \frac{\tan \beta }{\tan \alpha }=c $$

## Abbreviations

ONH: Optic nerve head
SD-OCT: Spectral-domain optical coherence tomography
